# A novel chicken infectious anemia virus vaccine candidate: complete attenuation, strong immunogenicity, and a built-in DIVA marker

**DOI:** 10.1016/j.psj.2026.107139

**Published:** 2026-05-15

**Authors:** HyeSoon Song, HyeonSu Kim, Moon Her, HyeRyoung Kim

**Affiliations:** Avian disease division, Animal and Plant Quarantine Agency, 177 Hyeoksin 8-ro, Gimcheon-si, Republic of Korea

**Keywords:** Chicken infectious anemia virus, Live‑attenuated vaccine candidate, P100, Immunogenicity, Multiplex PCR, Differentiation

## Abstract

Chicken infectious anemia virus (CIAV) is a globally important immunosuppressive pathogen of poultry, causing anemia, impaired immunity, and production losses. Current live-attenuated vaccines have practical limitations: incomplete attenuation restricts safe use to older birds (e.g. ≥ 6 weeks), while over-attenuation can reduce in-vivo replication and manufacturable vaccine titers. To address these gaps, we serially passaged the virulent Korean field strain 17AD008 in MDCC-MSB1 cells for 200 generations, generating two attenuated derivatives, P45 and P100. Pathogenicity and replication were evaluated in SPF chicks, followed by a homologous immunization-challenge model. Early host responses were analyzed by thymic immune-related gene expression profiling, and a novel multiplex PCR assay targeting a unique single nucleotide polymorphism (SNP; A750G) in the overlapping VP2/VP3 coding region of P100 was developed as a molecular DIVA (Differentiating Infected from Vaccinated Animals) tool. P100 was fully attenuated yet retained replication in lymphoid tissues, unlike P45, which caused anemia and thymic atrophy. Notably, P100 induced stronger early antiviral and pro-inflammatory transcriptional responses than parental strain without associated pathology. In homologous challenge studies, P100 administered at four weeks prevented thymic lesions and anemia, substantially curtailed cloacal virus shedding, and elicited higher CIAV-specific antibody titers. The multiplex PCR assay targeting the VP2/VP3 SNP (A750G) unambiguously distinguished P100 from field isolates, with 100% sensitivity and specificity. In conclusion, these data identify P100 as a DIVA-compatible live-attenuated CIAV vaccine candidate that combines complete attenuation, robust immunogenicity and transmission-blocking potential, warranting field evaluation for cross-genotype protection and large-scale production stability.

## Introduction

Chicken infectious anemia virus (CIAV), a member of the genus *Gyrovirus* in the family *Anelloviridae*, is a small, non-enveloped, icosahedral virus with an ∼2.3 kb circular, single-stranded, negative-sense DNA genome (Rosario et al., 2017; Liu et al., 2022). This genome encodes three overlapping open reading frames, VP1, the capsid protein essential for replication and infectivity, VP2, a scaffold protein that contributes to viral assembly and cytopathic effects, and VP3 (apoptin), which induces apoptosis in lymphoblastoid T cells and myeloid lineage cells, thereby driving immunosuppression (Noteborn et al., 1994; 1998; Iwata et al., 1998; Feng et al., 2020). The immunosuppressive effects of CIAV are most pronounced in chicks under three weeks of age that lack maternally derived antibodies, leading to increased susceptibility to secondary and opportunistic infections (Quaglia et al., 2021; Fatoba et al., 2022; Wang et al., 2025). Such immunological deficits translate into elevated morbidity, mortality, and significant production losses in commercial poultry flocks (McIlroy et al., 1992).

To date, with no specific antiviral treatment available, prevention relies primarily on vaccination in combination with stringent biosecurity, particularly to block vertical transmission from breeders to progeny (Kaffashi et al., 2008; Li et al., 2022; Sreekala et al., 2024). Commercial CIAV vaccines are generally live-attenuated strains derived by prolonged serial passage of virulent field isolates in embryonated eggs or in Marek’s disease virus-transformed lymphoblastoid cells (MDCC-MSB1) (McNulty., 1991; Tseng et al., 2019). Classical examples include the Cux-1 strain, which showed reduced pathogenicity after 49 passages in cell culture (Bülow and Fuchs., 1986), strains attenuated following extensive MDCC-MSB1 passage (173-320 generations) (Todd et al., 1998; 2002), and Malaysian isolates partially attenuated after 60-123 passages (Chowdhury et al., 2003). However, serial passage presents practical trade-offs: incomplete attenuation can leave residual virulence that constrains the minimum safe vaccination age, while excessive passage may impair in-vivo replication and reduce immunogenicity or manufacturability by limiting achievable vaccine titers. Consequently, several licensed CIAV vaccines are recommended only for older breeders (e.g. ≥ 6 weeks) (Wang et al., 2025). Together, these limitations highlight the need for vaccines that are fully attenuated yet retain sufficient replicative capacity for robust, durable immunity and scalable production. In our previous surveillance of Korean poultry flocks, we identified and propagated multiple CIAV field strains using MDCC-MSB1 cells. Among these, the 17AD008 strain displayed exceptionally high viral titers, stable replication kinetics, and strong adaptation to cell culture (Song et al., 2024), making it an attractive candidate for attenuation and vaccine development.

Here, we subjected 17AD008 to extended serial passage (up to 200 passages) in MDCC-MSB1 cells and generated two attenuated derivatives, P45 and P100, for vivo evaluation. We compared their pathogenicity, replication competence, and immunogenicity with the parental virulent strain and the commercial 26P4 vaccine. To explore molecular correlations of attenuation and protective efficacy, we performed immune-related gene expression profiling of immune-related genes in the thymus during early infection. Finally, to support future field application, we developed a strain-specific multiplex PCR assay enabling rapid and unambiguous differentiation of P100 from wild-type CIAV. Collectively, this study aimed to develop and characterize a novel CIAV vaccine candidate originated from Korean field strain, with an emphasis on both immunological performance and practical applicability.

## Materials and methods

### Ethical approval

All animal experiments were approved by the Animal Ethics Committee of the Animal and Plant Quarantine Agency (Approval ID:2023-714), and were conducted in strict accordance with institutional and national guidelines. One-day-old specific-pathogen-free (SPF) chicks and 4-week-old SPF chickens were assigned to treatment groups and housed in separate isolators under controlled temperature and humidity. Animals involved in this study were humanely euthanized by CO₂ inhalation with a target chamber concentration of ≥ 80% (v/v), maintained for ≥ 2 min after apnea (death confirmed by absence of corneal reflex and heartbeat).

### Viruses and vaccine strains

The wild-type (WT) 17AD008 was originally isolated from 9-week-old Korean native chickens and genetically characterized in a previous study (GenBank accession no. MW091338; Song et al., 2024). For attenuation, WT 17AD008 was serially passaged up to 200 times (P1–P200) in MDCC-MSB1 cells (1 × 10⁵ cells/mL). Cultures were monitored daily for cytopathic effects (CPE), and supernatants were harvested 3-4 days post-infection for subsequent passages and genomic analyses. The complete genomic nucleotide and deduced amino acid sequences of WT 17AD008 and its passage derivatives (P45, P100, P120, P150, P180, and P200) were determined as described previously (Song et al., 2024). A commercial live attenuated CIAV vaccine (Nobilis® CAV P4; MSD Animal Health, USA) was included for comparative evaluation.

### Pathogenicity assessment

To assess whether serial passage attenuates the virulent WT 17AD008, the virus was propagated in MDCC-MSB1 cells for up to 200 passages. These two derivatives, P45 and P100, together with the WT 17AD008 strain, were used for experimental infection of one-day-old chicks.

**Fig. 1:**
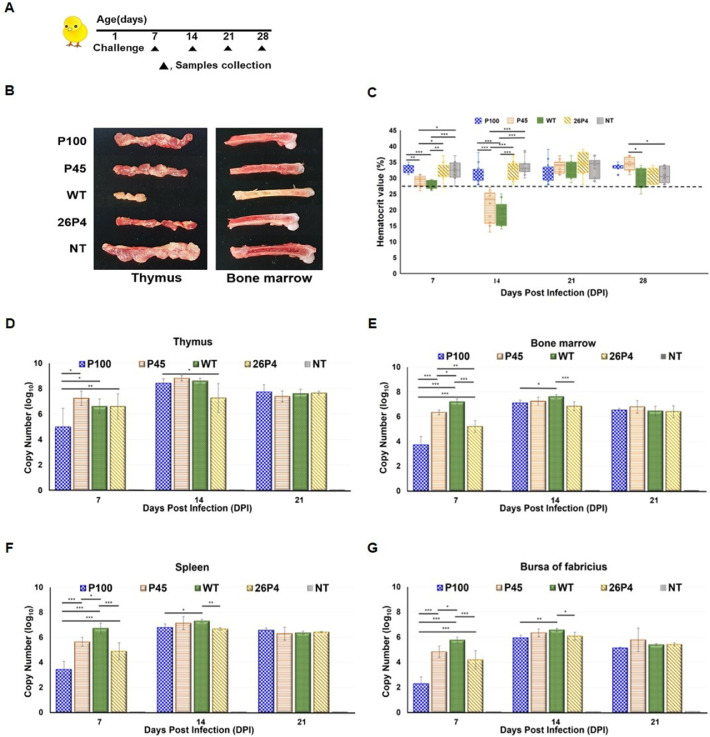
**Attenuation of CIAV through extended serial passage while preserving replication competence.** (A) Pathogenicity of WT 17AD008 and its derivatives in one-day-old SPF chicks. Experimental groups including WT 17AD008, P45, P100, 26P4, and negative control (NT) (n = 18 per group). (B) Gross thymus and bone marrow lesions at 14 dpi (n = 3 per group): severe thymic atrophy and yellow marrow in WT 17AD008; normal appearance in P100, 26P4, and NT; intermediate changes in P45. (C) PCV (n = 15 per group at 14 dpi): WT 17AD008 and P45 developed anemia (mean PCV < 27%), whereas P100 and 26P4 remained normal. (D-G) Viral DNA loads in lymphoid tissues (n = 3 per group at each time point): high genome copies from 7 to 21 dpi in all inoculated groups, indicating preserved replication. P100 retained replication capacity without anemia or gross lesions. Error bars represent the mean ± SD. Statistical significance was determined by one-way ANOVA with Tukey’s multiple comparison test (*p < 0.05, **p < 0.01, ***p < 0.001).

90 one-day-old SPF chicks were randomly divided into five groups (n = 18 per group): P100, P45, WT 17AD008, 26P4 (commercial vaccine strain), and negative control (NT, RPMI-1640 medium). Experimental groups were inoculated intramuscularly with 10^3.0^ TCID₅₀/0.1 mL of P100, P45, or WT 17AD008, and with 26P4 at the same dose (10^3.0^ TCID₅₀/0.1 mL) recommended by the manufacturer. The day of inoculation (one day of age) was designated as 0 days post infection (dpi), while control birds received an equivalent volume of RPMI 1640 medium (Fig. 1A).

Clinical signs were monitored daily. At 7, 14, and 21 dpi, three birds per group were euthanized by CO_2_ inhalation for collection of thymus, bone marrow, spleen, and bursa of Fabricius. The size of thymus and color of the femur bone marrow was recorded at necropsy.

Packed cell volume (PCV) until 28 dpi was assessed weekly by centrifuging whole blood in microhematocrit capillary tubes at 1200 × g for 5 min. A PCV below 27.0% was considered indicative of anemia.

Viral replication in tissues was quantified by real-time PCR as previously described (Song et al., 2024).

### Gene expression analysis

15 one-day-old SPF chicks were randomly assigned to three groups (n = five per group): P100, WT 17AD008, and negative control (NT, RPMI-1640). Experimental groups received intramuscular inoculation with 10^6.0^TCID₅₀/0.1 mL of the respective virus. At 3 dpi, all chicks were euthanized by CO_2_ inhalation and thymus tissues collected. Total RNA was extracted using TRIzol reagent (Thermo Fisher Scientific, USA), and cDNA was synthesized from 1 µg RNA using the SuperScript III First-Strand Synthesis System (Thermo Fisher Scientific, USA) according to the manufacturer’s protocol. The cDNA was diluted 1:10 and used for quantitative real‐time PCR in 20 µL reactions containing SYBR Green Master Mix (Thermo Fisher Scientific, USA) and gene‐specific primers targeting IL-18, IFN-γ, IL-1β, IFN-α, IFN-β, GADD45γ, and GAPDH (Table 1, Supplementary Fig. 1). Amplification was performed on a QuantStudio 5 Real-Time PCR System (Applied Biosystems, USA) under the following conditions: 50°C for 10 min for reverse transcription, 95°C for 2 min for initial denaturation and polymerase activation, followed by 40 cycles of 95°C for 1 sec for denaturation and 60°C for 20 sec for annealing and extension, and finally a melt-curve analysis to verify specificity of the amplified products. Relative expression levels were normalized to GAPDH and calculated using the 2^-ΔΔCt^ method relative to NT. Viral genome copy number in the same tissue was quantified by real-time PCR, as previously described (Song et al., 2024).

**Table 1:** Primer sets used for SYBR Green real-time PCR of host gene expression and multiplex PCR for DIVA assay.

Purpose	Target gene	Primer	Sequences 5′-3′	Amplicon size (bp)
Host Gene expression	IL-1β	Forward	TCGCCTGGATTCTGAGCAC	177
		Reverse	CAAAAACCTCCTCCAGGAGCG	
	IL-18	Forward	AGTGGAATGTACTTCGACATTCAC	148
		Reverse	TCCTTCCCTAAATCGAACAACC	
	IFN-α	Forward	GACATCCTTCAGCACCTCTTCAA	196
		Reverse	GCTGAAGTGTTTTTTGATGGTGAGG	
	IFN-β	Forward	CAATGCTTCGTAAACCAAGGCAC	189
		Reverse	ATCCATTGTATGAGTGTGTCCACG	
	IFN-γ	Forward	AGGATCATACTGAGCCAGATTGT	138
		Reverse	TTCACCTTCTTCACGCCATCAG	
	GADD45γ	Forward	GCAGATCCACTTCACCCTTATC	150
		Reverse	GGTTCGTGATGAGAATGCAGT	
	GAPDH	Forward	GAAAGTCATCCCTGAGCTGAATG	185
		Reverse	CTGGTCCTCTGTGTATCCTAGG	
DIVA	VP2/VP3	Forward	GATCCGGATTGGTATCGCTG	425/208
		Reverse	GGTTGTGAAAGGCCTGGCTAA	
		Reverse	CTTTTAGCTCGCTTACCGCCTACT	

### Immunization-challenge study

In the immunization-challenge study, SPF chickens were immunized with P100 or 26P4 at 4 weeks of age and challenged with WT 17AD008 two weeks later (6 weeks of age) (Fig. 3A).

80 4-week-old SPF chickens were allocated into four groups (n = 20 per group): P100, 26P4 (commercial vaccine strain), WT 17AD008, and negative control (NT, RPMI-1640 medium). Chickens in the P100 and 26P4 groups were intramuscularly immunized with 10^3.0^ TCID₅₀/0.1 mL of their respective viruses. The day of immunization was designated 0 days post challenge (dpc). At 6 weeks of age, all chickens in P100, 26P4, and WT groups were challenged intramuscularly with 10^7.5^TCID_50_/0.1 mL of WT 17AD008 strain, while the NT group received the same volume of RPMI 1640.

Three chickens from each group were euthanized by CO_2_ inhalation at 7-day intervals after challenge for tissue collection (thymus, spleen, bursa of Fabricius, and cecal tonsils). The size of thymus was assessed at 14 dpc.

Blood and cloacal swabs were taken from all remaining chickens at 0, 7, 14, 21, 28, and 35 dpc. PCV and viral loads were determined as described in the pathogenesis study.

Serum samples were used to monitor the development of CIAV-specific antibodies by means of a commercially available CIAV ELISA kit (BioChek, USA). All chicks were confirmed to be seronegative for CIAV-specific antibodies at the time of inoculation.

### Multiplex PCR assay design and validation

Sequence alignment for the complete sequences identified a unique single nucleotide polymorphism (SNP) at nucleotide position 750 nt in the P100 strain when compared with global CIAV reference sequences from the NCBI database (Supplementary Fig. 2), which was subsequently targeted for primer design (Table 1) using OligoAnalyzer (Integrated DNA Technologies, USA). The multiplex PCR assay was carried out in a 20 µL reaction containing 2 µL of template DNA, 1 µL each of CIAVF and CIAVR primers (4 pmol/µL), 1 µL of CIAVR1 primer (12 pmol/µL), and BlackPCR Multiplex PCR Premix (Ventech Science, Korea). Thermal cycling conditions consisted of an initial denaturation at 94°C for 5 min, followed by 40 cycles of 94°C for 50 sec, 59°C for 50 sec, and 72°C for 50 sec, with a final extension at 72°C for 7 min.

Specificity was confirmed using genomic DNA from P100, 26P4, WT 17AD008, and ten additional field isolates (17AD007, 09AD367, 19AQ026, 10AQ095, 09AD354, 18R059, 09AD301, 19AD011, 08AQ017A, 18R001). Assay performance was validated using 32 cloacal swabs collected during the immunization–challenge study (16 P100-immunized/challenged, 8 26P4-immunized/challenged, and 8 WT-challenged).

### Statistical analysis

Statistical analyses were performed using GraphPad Prism v5.0.1 (GraphPad Software, USA). For gene expression and other time-course data, differences among multiple groups at the same time point were evaluated by one-way analysis of variance (ANOVA) followed by Tukey’s multiple comparison test. A p-value < 0.05 was considered statistically significant, and the underlying raw data and detailed statistical outputs are provided in Supplementary Table 1.

## Results

### Attenuation through extended serial passage while preserving replication competence

WT 17AD008 was propagated in MDCC-MSB1 cells for up to 200 passages, while maintaining stable CPE and high viral titers (∼10^7.5^ TCID_50_/0.1 mL; data not shown). During this attenuation process, we monitored sequence changes in the *VP1* gene and identified three nucleotide positions (915, 1253, and 2161) that evolved progressively with increasing passage number. The WT 17AD008 carried an A, A, and T pattern at these sites, whereas intermediate passages (e.g., P45 and P73) showed mixed nucleotides, indicating heterogeneous viral populations. By passage 100 (P100), these positions had become fixed as G, T, and G, respectively, suggesting selection for a distinct *VP1* variant (Table 2, Supplementary Fig. 3A). These nucleotide changes corresponded to amino acid alterations in VP1 at positions 28 (R28), 141 (Q→L; Q141L), and 444 (Y→D; Y444D) (Supplementary Fig. 3B). We then compared the *in vivo* pathogenicity of WT 17AD008, P45, and P100 by inoculating one-day-old chicks with each virus.

**Table 2:** Nucleotide variations in the *VP1* gene of the WT 17AD008 strain during in vitro serial passaging up to passage 200.

	Nucleotide position						
	VP1						VP2/VP3
Virus (passage level)	915	1253	1300	1640	1696	2161	715
WT17AD008 (0)	A	A	A	C	A	T	A
P35 (35)	ㆍ	ㆍ	ㆍ	C/T	ㆍ	T/G	ㆍ
P45 (45)	A/G	ㆍ	ㆍ	C/T	ㆍ	T/G	ㆍ
P62 (62)	A/G	ㆍ	ㆍ	C/T	ㆍ	T/G	ㆍ
P73 (73)	A/G	A/T	ㆍ	C/T	ㆍ	T/G	ㆍ
P88 (88)	A/G	T	ㆍ	C/T	ㆍ	T/G	G
P100 (100)	G	T	ㆍ	C	ㆍ	G	ㆍ
P120 (120)	ㆍ	ㆍ	ㆍ	ㆍ	ㆍ	ㆍ	ㆍ
P137 (137)	ㆍ	ㆍ	G	ㆍ	ㆍ	ㆍ	ㆍ
P141 (141)	ㆍ	ㆍ	ㆍ	ㆍ	ㆍ	ㆍ	ㆍ
P150 (150)	ㆍ	ㆍ	ㆍ	ㆍ	ㆍ	ㆍ	ㆍ
P153 (153)	ㆍ	ㆍ	ㆍ	ㆍ	ㆍ	ㆍ	ㆍ
P160 (160)	ㆍ	ㆍ	ㆍ	ㆍ	ㆍ	ㆍ	ㆍ
P170 (170)	ㆍ	ㆍ	ㆍ	ㆍ	G	ㆍ	ㆍ
P180 (180)	ㆍ	ㆍ	ㆍ	ㆍ	ㆍ	ㆍ	ㆍ
P190 (190)	ㆍ	ㆍ	ㆍ	ㆍ	ㆍ	ㆍ	ㆍ
P200 (200)	ㆍ	ㆍ	ㆍ	ㆍ	ㆍ	ㆍ	ㆍ

WT 17AD008 caused depression, reduced mobility, and generalized weakness from 14 to 18 dpi, consistent with previous findings (Song et al., 2024). In contrast, P100-, P45-, and 26P4-inoculated birds remained clinically healthy. Especially, no mortality, clinical depression, or long-term growth suppression was observed in the P100 group throughout the 28-day observation period. Necropsy results showed that WT group exhibited marked thymic atrophy and a distinct conversion of red to yellow bone marrow, indicative of impaired hematopoiesis at 14 dpi. However, P100-, P45-, and 26P4-infected chicks showed comparable thymus size and bone marrow coloration, without the pathological changes observed in the WT group (Fig. 1B).

PCV values ranged from 28.0 - 38.0% in P100, 13.0 - 38.0% in P45, 14.0 - 37.0% in WT, 27.0 −39.0% in 26P4, and 27.5 - 39.0% in NT groups. At 14 dpi, both P45 and WT groups exhibited mean PCV < 27.0%, indicating severe anemia, which subsequently recovered; no anemia occurred in P100- or 26P4-immunized groups, comparable to NT controls (data are mean ± SD, Fig. 1C).

Viral DNA loads in the thymus, bone marrow, spleen, and bursa of Fabricius remained consistently high from 7 to 21 dpi in all inoculated groups, indicating that both attenuated and virulent strains replicated efficiently in lymphoid tissues, whereas no viral genome was detected in NT controls (data are mean ± SD, Fig. 1D-G).

### Robust immune activation without pathogenicity

**Fig. 2:**
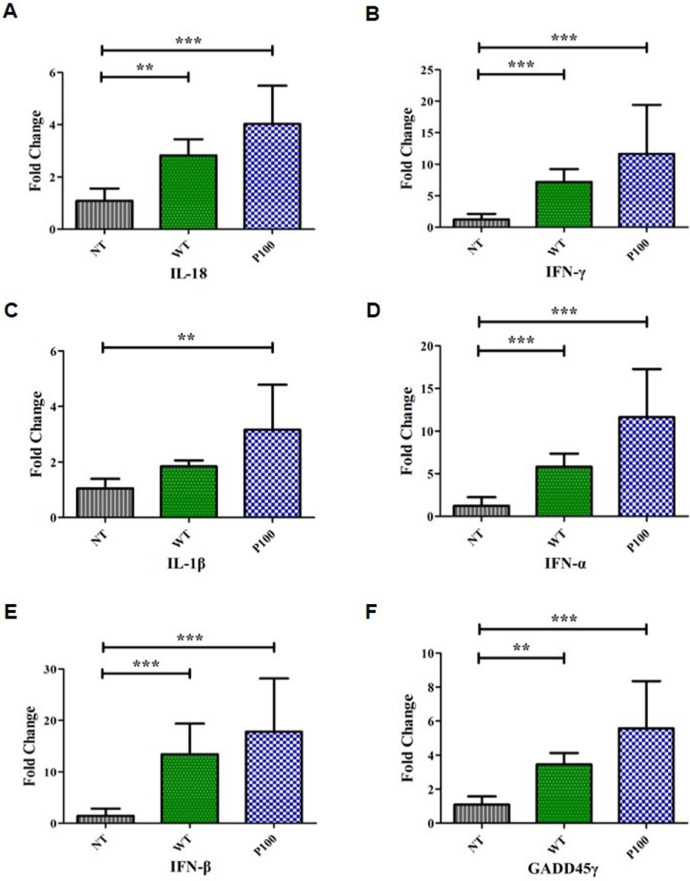
P100 induces robust cytokine responses without pathogenicity. Relative mRNA expression of (A) IL-18, (B) IFN-γ, (C) IL-1β, (D) IFN-α, (E) IFN-β, and (F) GADD45γ in thymus tissues at 3 dpi. Expression levels were presented as fold change relative to non-infected controls (normalized to *GAPDH*). Both WT 17AD008 and P100 infection significantly upregulated antiviral and inflammatory cytokines, with P100 eliciting stronger responses. Despite strong cytokine induction, no clinical signs or gross lesions were observed in the P100 group. Error bars represent mean ± SD (n = 5 per group). Statistical significance was determined by one-way ANOVA with Tukey’s multiple comparison test (*p < 0.05, **p < 0.01, ***p < 0.001).

At 3 dpi, CIAV genomes were readily detected in the thymus of all infected chicks (Supplementary Table 2). WT infection induced significant upregulation of IL-18 (2.83-fold), IFN-α (5.81-fold), IFN-β (13.40-fold), and GADD45γ (3.46-fold) relative to controls (p < 0.05). P100 infection elicited even greater induction of IL-18 (4.03-fold), IFN-γ (11.66-fold), IFN-α (11.62-fold), IFN-β (17.80-fold), and GADD45γ (5.57-fold) (data are mean ± SD, Fig. 2A-F). For IL-1β, no statistically significant difference was observed between the WT and NT groups (p > 0.05), whereas chicks inoculated with P100 showed a 3.16-fold increase. Notably, this heightened antiviral and inflammatory activation occurred in the absence of clinical signs or gross lesions in the P100 group.

### Superior protection compared with the current commercial vaccine in 4-week-old chickens

**Fig. 3:**
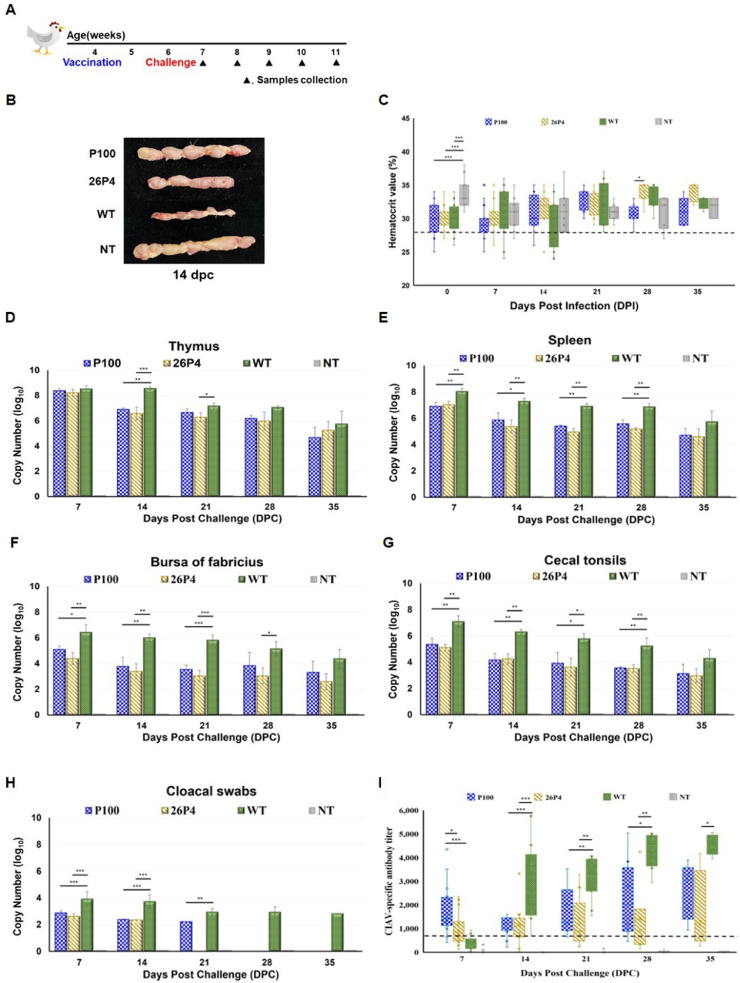
**Protective efficacy of the P100 vaccine candidate in 4-week-old chickens.** (A) Immunization-challenge design (n = 20 per group): SPF chickens were immunized at 4 weeks of age and challenged with WT 17AD008 two weeks later. (B) Gross thymus lesions at 14 dpc (n = 3 per group): severe atrophy in WT 17AD008, mild in 26P4, none in P100. (C) PCV at 14 dpc (n = 17 per group): all P100- and most 26P4-immunized birds remained above the anemia threshold (27%), while 28.6% of WT controls fell below. (D-G) Viral genome loads in organs (n = 3 per group at each time point): highest in WT 17AD008, reduced in vaccinated groups. (H) Viral shedding in cloacal swabs (n = all remaining birds per group at each time point): detected up to 21 dpc in P100, 14 dpc in 26P4, and throughout in WT controls. (I) CIAV-specific antibodies (n = all remaining birds per group at each time point): highest in WT controls; P100 > 26P4; NT remained seronegative. These findings indicate that P100 confers strong protection against virulent CIAV. Error bars represent the mean ± SD. Statistical significance was determined by one-way ANOVA with Tukey’s multiple comparison test (*p < 0.05, **p < 0.01, ***p < 0.001).

At 14 dpc, WT-challenged, non-immunized controls displayed severe thymic atrophy, while P100-immunized birds exhibited no gross thymic lesions. Birds immunized with 26P4 exhibited only mild thymic atrophy, less severe than in WT controls but more pronounced than in the P100 group (Fig. 3B).

PCV values remained above the anemia threshold in all P100- and most 26P4-immunized birds, whereas 28.6% (4/14) of WT-challenged birds fell below 27.0% at 14 dpc (data are mean ± SD, Fig. 3C).

Viral genome loads in lymphoid tissues revealed a gradual decline in all challenged groups but remained detectable until 35 dpc. WT controls consistently harbored the highest viral loads, whereas P100- and 26P4-immunized groups maintained substantially lower levels across all tissues (data are mean ± SD, Fig. 3D-G). Analysis of cloacal swabs showed that, in the P100 group, viral shedding occurred only sporadically and was reduced to low-level detection in just two birds at 21 dpc, consistent with near-complete viral clearance. In contrast, shedding in the 26P4 group persisted until 14 dpc, whereas WT controls continued to shed virus throughout most of the observation period (data are mean ± SD, Fig. 3H). The positive detection rates were 81.3% at 7 dpc and 30.8% at 14 dpc in the 26P4 group; 58.8% at 7 dpc, 28.6% at 14 dpc, and 18.2% at 21 dpc in the P100 group; and 100.0% from 7 to 21 dpc, 50.0% at 28 dpc, and 60.0% at 35 dpc in the WT group.

From 14 dpc onward, WT controls consistently exhibited the highest CIAV-specific antibody titers, reflecting strong seroconversion after natural infection. Among vaccinated groups, P100 elicited markedly higher antibody titers than 26P4, indicating superior immunogenicity. As expected, all NT birds remained seronegative throughout the study (data are mean ± SD, Fig. 3I).

### Development of a molecular DIVA tool

**Fig. 4:**
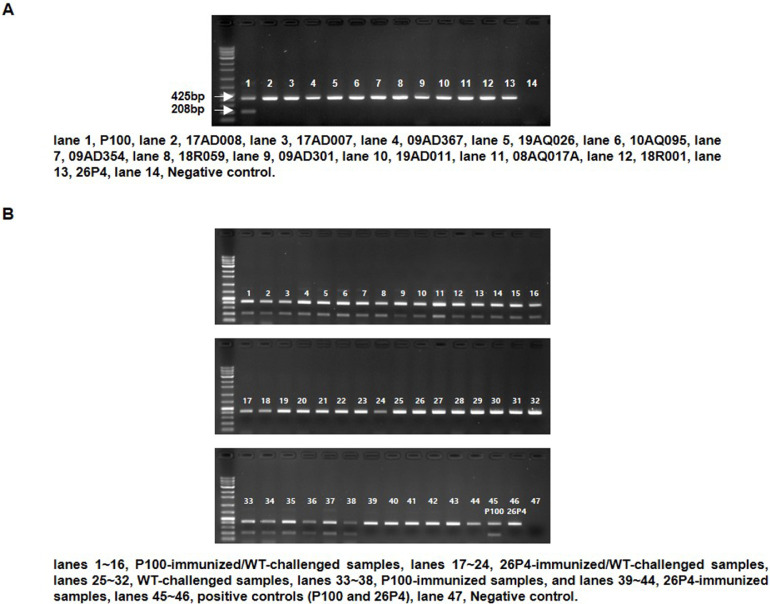
**Development and validation of a P100-specific molecular DIVA assay.** (A) Multiplex PCR using universal and P100-specific primers generated two amplicons (425 bp for WT CIAV, 208 bp for P100). The 208 bp fragment was detected exclusively in P100 (lane 1) and was absent in 26P4 (lane 13) and field isolates (lanes 2-12); lane 14 served as the negative control. (B) Validation with cloacal swabs showed dual bands in P100-immunized/challenged samples (lanes 1–16), whereas only the 425 bp band was detected in 26P4-immunized/challenged (lanes 17–24) and WT-challenged groups (lanes 25–32). Dual-band amplification was also observed in P100-immunized samples (lanes 33-38), whereas single-band amplification was detected in 26P4-immunized samples (lanes 39-44). Positive controls (lanes 45-46) and the negative control (lane 47) are also shown. The assay demonstrated 100% sensitivity and specificity with no cross-reactivity.

Comparative genomic analysis revealed that a unique SNP (A750G), located in the overlapping VP2/VP3 coding region, was present only in P100. Using this marker, we developed a multiplex PCR assay that consistently generated two distinct amplicons: a 425 bp fragment for wild-type CIAV and a 208 bp fragment for P100 (Fig. 4A, lane 1). No P100-specific amplification was observed with the 26P4 vaccine strain or ten genetically diverse field isolates (Fig. 4A, lanes 2-13). Sanger sequencing confirmed 100% identity of all amplicons with the corresponding reference genomes (data not shown).

Diagnostic performance was further validated using 32 cloacal swabs from the immunization-challenge study. Both 425 bp and 208 bp fragments were detected in all P100-immunized/challenged samples (Fig. 4B, lanes 1-16). By contrast, only the 425 bp wild-type fragment was amplified in 26P4- immunized/challenged and WT-challenged samples (Fig. 4B, lanes 17-24, and 25-32). In addition, cloacal swab samples collected from birds immunized with P100 showed dual-band amplification, whereas samples from the 26P4-immunized group exhibited only single-band amplification corresponding to the wild-type fragment (Fig. 4B, lanes 33-38 and 39-44). These results demonstrate that the multiplex PCR assay achieved 100% sensitivity and specificity, with no cross-reactivity to non-target CIAV strains.

## Discussion

CIAV, first identified in Japan in 1979, is now recognized as a globally important immunosuppressive pathogen in poultry (Yuasa et al., 1979; Techera et al., 2021; Wang et al., 2022). Outbreaks can lead to substantial economic losses due to anemia, immunosuppression, and increased mortality, as documented in both layer and broiler flocks (McIlroy et al., 1992; Zeng et al., 2023). Moreover, since CIAV is highly resistant to environmental inactivation and difficult to eradicate once introduced (McNulty., 1991; Miller et al., 2005), vaccination against CIAV remains the most practical and sustainable control strategy.

Most commercial vaccines, including 26P4, Del-Ros, and Cux-1, were generated by serial passage of virulent field isolates in embryonated eggs or cell culture (Song et al., 2024; Wang et al., 2025). Although these vaccines help reduce vertical transmission, many retain residual pathogenicity in young birds and are therefore recommended only for older breeders (e.g., 9-15 weeks of age in the United States) (Gimeno and Schat., 2018; Wang et al., 2025). This creates a critical window, from the waning of maternally derived antibodies until the recommended vaccination age, during which chickens are vulnerable to CIAV infection and its secondary consequences. Consequently, there is a need for vaccine candidates that can be safely administered earlier to establish protective immunity sooner. In addition, recent epidemiological studies suggest that CIAV dissemination may occur through international poultry trade and migratory bird movements, contributing to the spread of genetically divergent strains across borders (Techera et al., 2021; Xu et al., 2023). Such findings highlight the importance of continuous surveillance of imported breeders and wild bird populations, as well as the need for vaccines to ensure effective protection under field conditions. In Korea, CIAV was first detected in 1989 (Kim et al., 2010), and an imported attenuated vaccine such as 26P4 has been administered in breeders since 2005. In this study, we developed the first CIAV vaccine candidate derived from a Korean field isolate, the WT 17AD008 strain, by serially passaging the virus up to 200 times in MDCC-MSB1 cells. Throughout this phase, progressive serial passage led to the fixation of three amino acid substitutions (R28, Q141L, and Y444D) in the VP1 protein, indicating a gradual shift from a heterogeneous viral population to fixed mutations at these specific sites. On this basis, P45 and P100 were selected as representative intermediate and advanced passage levels. These specific passages exhibited clearly distinguishable VP1 mutation patterns while retaining robust replication in MDCC-MSB1 cells and lymphoid organs, thereby providing rational candidates for subsequent *in vivo* pathogenicity and vaccine evaluation. In addition, five back-passage experiments in SPF chicks, performed according to European Pharmacopoeia guidelines, confirmed that three nucleotide positions (915, 1253, and 2161) remained stably fixed as G, T, and G, respectively (Supplementary Fig. 4). This *in vivo* stability, together with the preserved replication, further supports the suitability of P100 as a live attenuated CIAV vaccine candidate.

Pathogenicity testing revealed that P100 was completely attenuated, causing no clinical signs or gross lesions, whereas P45 showed only partial attenuation. These results demonstrate that P100 fulfills the fundamental requirement for a live attenuated vaccine: loss of virulence without loss of replication capacity. Our data further indicate that P100 has clear immunological advantages over WT 17AD008, showing higher thymic expression of IL-18, IFN-γ, type I interferons (IFN-α/β), IL-1β and GADD45 family members. IL-18 is well known as a prototypical Th1-inducing, pro-inflammatory cytokine that promotes IFN-γ production by T and NK cells and supports cell-mediated antiviral immunity (Takeda et al., 1998; Nakanishi et al., 2001; Alboni et al., 2010), while type I interferons and IL-1β further enhance antiviral programs and help link innate signals to T-cell responses (Dinarello, 1999; Pyrillou et al., 2020). Although most of the mechanistic work dissecting the cooperation between IL-18, type I interferons, and IL-1β has been performed in mammalian systems, similar functional roles for IL-18, IFN-α/β, and IL-1β in shaping early antiviral and vaccine-induced immunity have also been reported in chickens, suggesting that analogous pathways may operate in our model.

In chickens, IL-18 has repeatedly been shown to act as a potent vaccine adjuvant, enhancing IFN-γ production, antibody responses, and cell-mediated immunity against various viral pathogens, including Newcastle disease virus (Hung et al., 2010), avian influenza virus H9N2 (Chen et al., 2011), and infectious bursal disease virus (Li et al., 2013), which is consistent with the enhanced protection observed in the P100-immunized groups. Moreover, the upregulation of Gadd45γ in P100-immunized birds is noteworthy, because Gadd45γ has been implicated in IL-18-driven Th1 differentiation and cytokine-induced IFN-γ production in mammalian systems (Schmitz, 2013; Ma et al., 2025). Although this mechanism has not yet been directly demonstrated in chickens, our findings are consistent with a scenario in which P100 primes a more effective Th1-biased antiviral response. Taken together, these observations suggest that the IL-18- and interferon-linked responses elicited by P100 are not merely correlative but may actively contribute to the superior protective efficacy of this attenuated CIAV vaccine candidate, even though the precise mechanistic contribution of each pathway remains to be clarified. Such an immune profile suggests that P100 achieves the dual goals of a live vaccine: robust immune activation and complete safety. Compared with a previous study that observed only modest cytokine induction at later stages of infection (Giotis et al., 2015), our early-phase analysis highlights P100’s ability to trigger rapid innate responses, potentially accelerating downstream adaptive immunity.

In the immunization-challenge model, P100 provided improved protection against homologous challenge at four weeks of age, largely preventing thymic atrophy, anemia, and sustained viral replication. By comparison, the commercial 26P4 vaccine afforded measurable but less complete protection under the same conditions. This difference may, at least in part, reflect the fact that 26P4 was evaluated in birds younger than the age recommended by the manufacturer. P100 demonstrated efficacy in 4-week-old chickens, supporting its potential to confer earlier protective immunity under field conditions. Importantly, P100 significantly reduced cloacal viral shedding, limiting it to 21 dpc, whereas wild-type controls shed virus continuously. Previous studies have reported that lymphoid tissues are the primary targets for CIAV infection and that CIAV can persist in thymic lymphocytes, where it cannot be effectively neutralized by antibodies (Markowski-Grimsrud et al., 2003; Tongkamsai et al., 2019a; Song et al., 2024). Continuous dissemination of virus from the thymus in the setting of an insufficient antibody response, together with the release of infected thymic lymphocytes into the circulation, is thought to drive increased viral distribution to other tissues (Tongkamsai et al., 2019b). Once disseminated, CIAV can replicate in secondary tissues and be transmitted both horizontally through contact and vertically via hatching eggs. Therefore, the marked reduction in viral shedding observed in the P100 group has important implications for limiting horizontal transmission under field conditions. Furthermore, CIAV-specific antibody titers followed the hierarchy WT > P100 ≥ 26P4. Although titers in P100-immunized birds remained lower than those observed in WT-infected controls, they were significantly higher than those induced by 26P4, likely reflecting closer antigenic homology between P100 and circulating Korean strains. This indicates that P100 can enhance humoral immunity beyond existing commercial vaccines while avoiding the pathological consequences of natural infection. Comparable observations were reported by Liu et al., who demonstrated that a DNA prime-protein boost vaccination strategy against CIAV elicited robust humoral and cellular immune responses, significantly reduced viral loads, and mitigated lymphoid pathology (Liu et al., 2022). However, unlike recombinant or prime-boost approaches, P100 achieves these outcomes as a live attenuated vaccine derived from a field isolate, combining complete attenuation with strong immunogenicity.

Furthermore, by leveraging a unique SNP that became fixed as G by passage 88 during serial passaging (Table 2) and is located at nucleotide position 750 nt in the overlapping VP2/VP3 coding region of P100, we developed a molecular DIVA assay to distinguish this vaccine candidate from field strains. This assay demonstrated 100% sensitivity and specificity, enabling reliable post-vaccination differentiation in field samples. This design is consistent with successful precedents in veterinary vaccinology, with avian influenza vaccines utilizing heterologous neuraminidase or NS1-deletion strategies (Capua et al., 2007; Wang et al., 2019), and lumpy skin disease virus programs implementing duplex qPCR and marker-based ELISA (Haegeman et al., 2023; Nokhwal et al., 2025). By aligning with these approaches, our P100-specific molecular DIVA assay offers a modern tool for outbreak tracing, surveillance, and vaccine program monitoring. The ability to pair a live vaccine with a built-in DIVA marker is particularly valuable for CIAV, where immunosuppressive effects complicate field diagnosis and surveillance, and positions P100 as a forward-looking tool for integrated disease management. Together with the demonstrated *in vivo* stability of the A750G marker, this supports the use of the VP2/VP3-based SNP as a robust molecular DIVA target for P100 (Supplementary Fig. 4).

In summary, P100 satisfies the key requirements of an optimal live attenuated CIAV vaccine, including (i) complete attenuation with preserved replication capacity, (ii) strong induction of innate and adaptive immunity, (iii) prevention of clinical disease and lymphoid pathology, (iv) reduced viral shedding, and (v) incorporation of a DIVA marker for surveillance. As the first CIAV vaccine candidate derived from a Korean field strain, P100 represents both an immunologically superior and strategically rational advance. Future field evaluations should be assessed to confirm the cross-protection against heterologous strains, large-scale production stability, long-term safety, and maternal antibody interference, but the present findings strongly support P100 as a promising candidate for practical application in CIAV control.

## Funding

This study was supported by grants from the 10.13039/501100008771Animal and Plant Quarantine Agency (Grant B-1543084-2025-28-01 and M-1543084-2023-25-01) and of the Ministry of Agriculture, Food and Rural Affairs of the Republic of Korea.

## Patents

The vaccine composition against chicken infectious anemia virus and the multiplex PCR primers for simultaneous detection of field and vaccine strains are patented or under patent application.

## CRediT authorship contribution statement

**HyeSoon Song:** Writing – original draft, Funding acquisition, Data curation, Conceptualization. **HyeonSu Kim:** Visualization, Validation, Methodology, Formal analysis. **Moon Her:** Writing – review & editing. **HyeRyoung Kim:** Writing – review & editing.

## Disclosures

The authors declare that they have no known competing financial interests or personal relationships that could have appeared to influence the work reported in this paper.

## Appendix Supplementary materials

Image, application 1

Image, application 2

Image, application 3

## Footnotes

Supplementary material associated with this article can be found, in the online version, at doi:10.1016/j.psj.2026.107139.
