# Supplementary material for: A novel chicken infectious anemia virus vaccine candidate: complete attenuation, strong immunogenicity, and a built-in DIVA marker
Source: Poult Sci. 2026 May 15;105(9):107139. doi: 10.1016/j.psj.2026.107139 (PMC13226897; doi:10.1016/j.psj.2026.107139)
Supplement: Supplementary file 1 [file mmc1.docx]

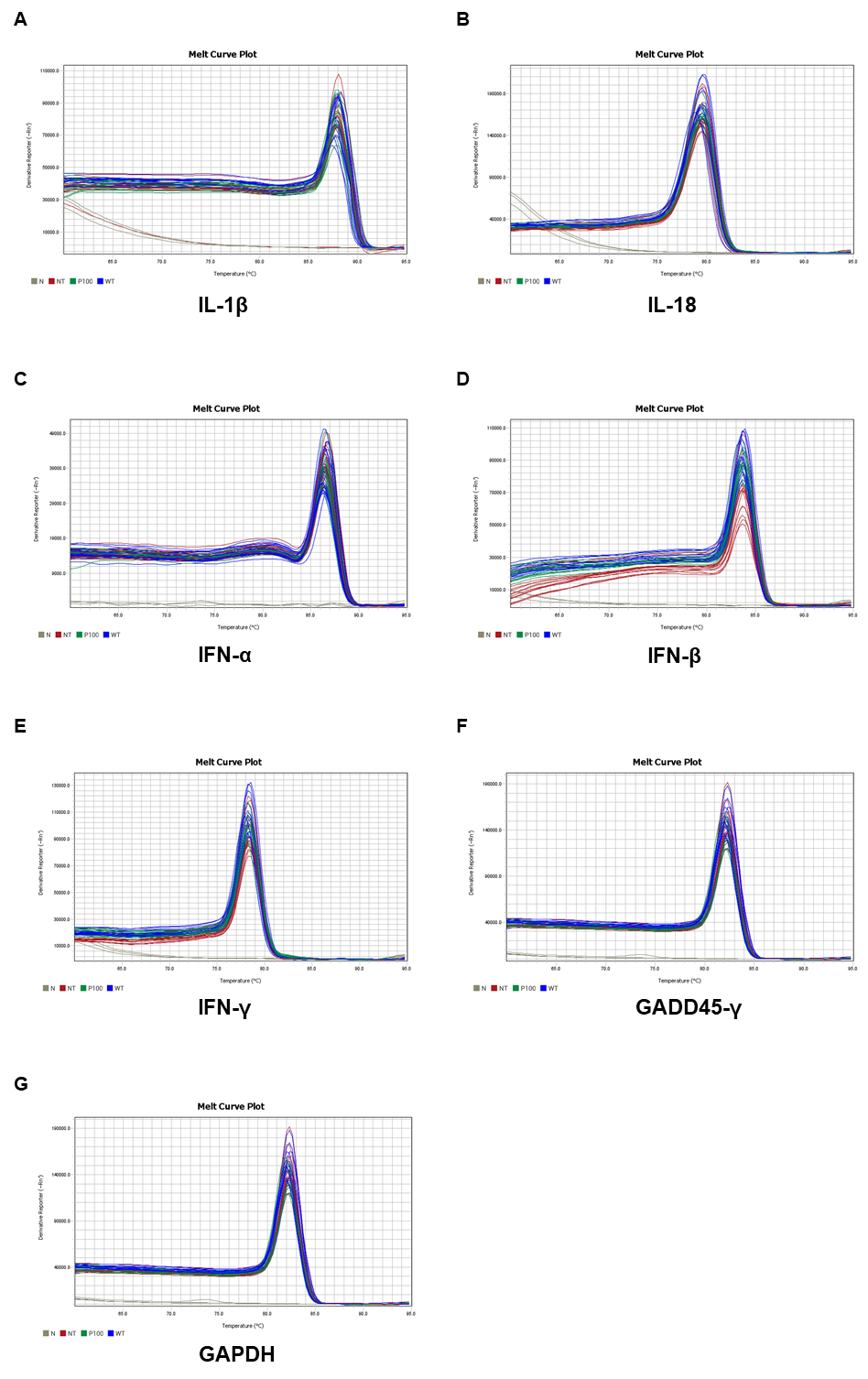


**Supplementary Fig. 1. Melting curve analysis of immune gene–specific primers used for relative expression quantification.** Shown are (A) IL-1β, (B) IL-18, (C) IFN-α, (D) IFN-β, (E) IFN-γ, (F) GADD45γ, (G) GAPDH. Each panel demonstrates a single, sharp melting peak, confirming primer specificity.


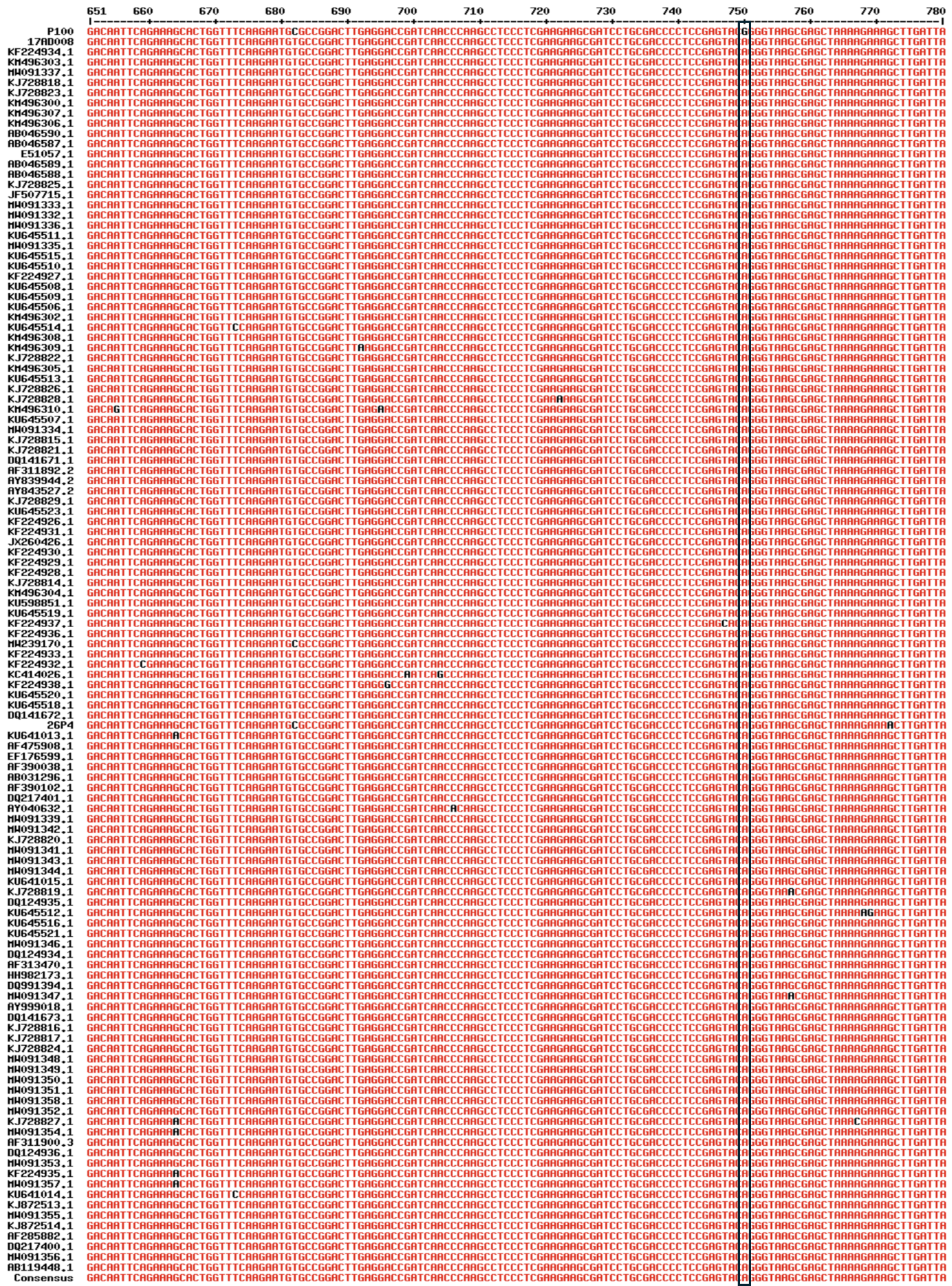


**Supplementary Fig. 2. Sequence alignment of nucleotide position 750 (nt 750) in the P100 strain compared with global CIAV reference sequences from the NCBI database. The conserved A750G single nucleotide polymorphism (SNP) is indicated.**


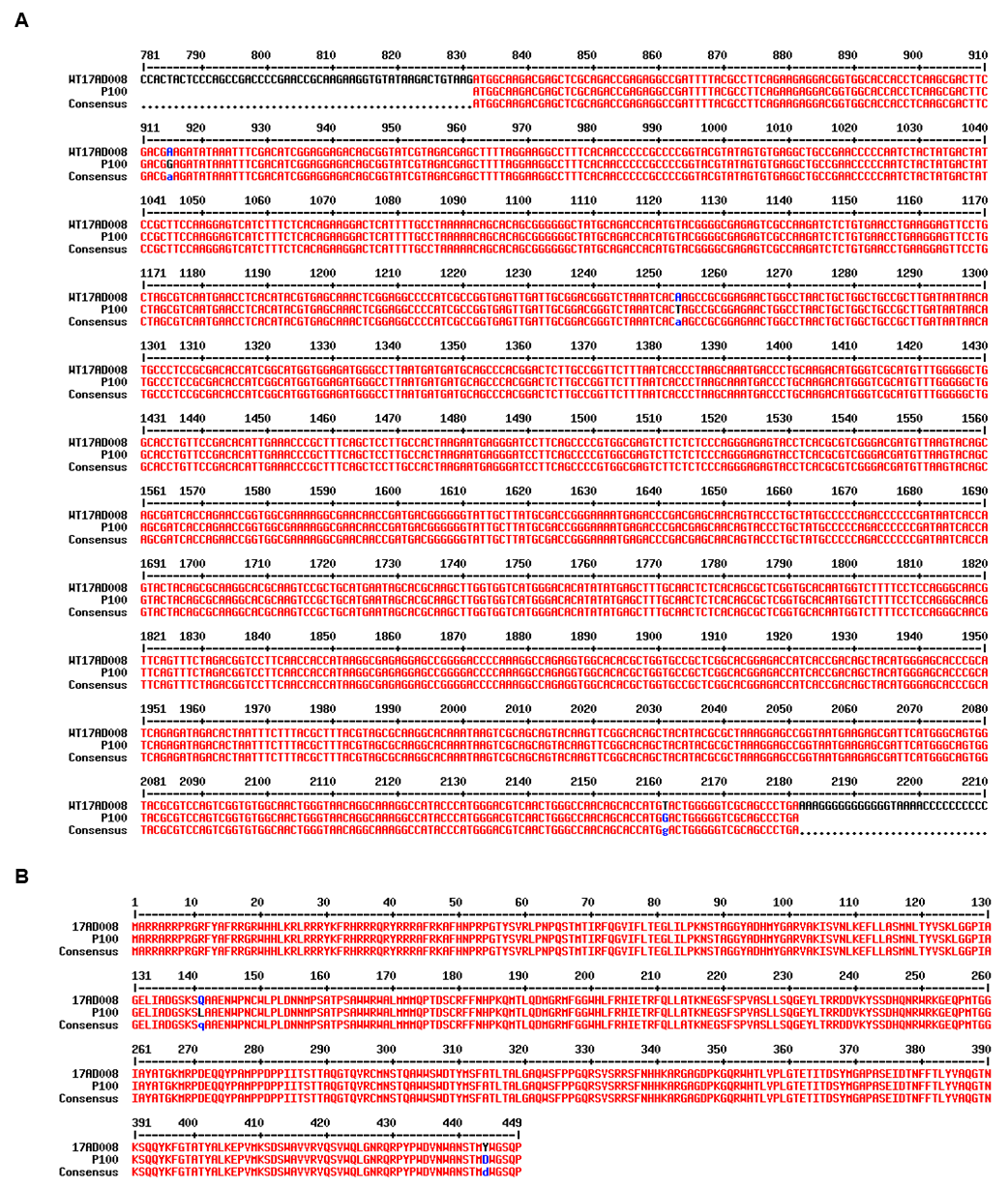


**Supplementary Fig. 3. Comparison of *VP1* sequences for WT 17AD008 and P100.**
(A) Alignment of *VP1* nucleotide sequences focusing on positions 915 (A → G), 1253 (A → T), and 2161 (T → G). (B) Alignment of *VP1* amino acid sequences highlighting residues 141(Q → L) and 444 (Y → D) corresponding to *VP1* changes in P100.


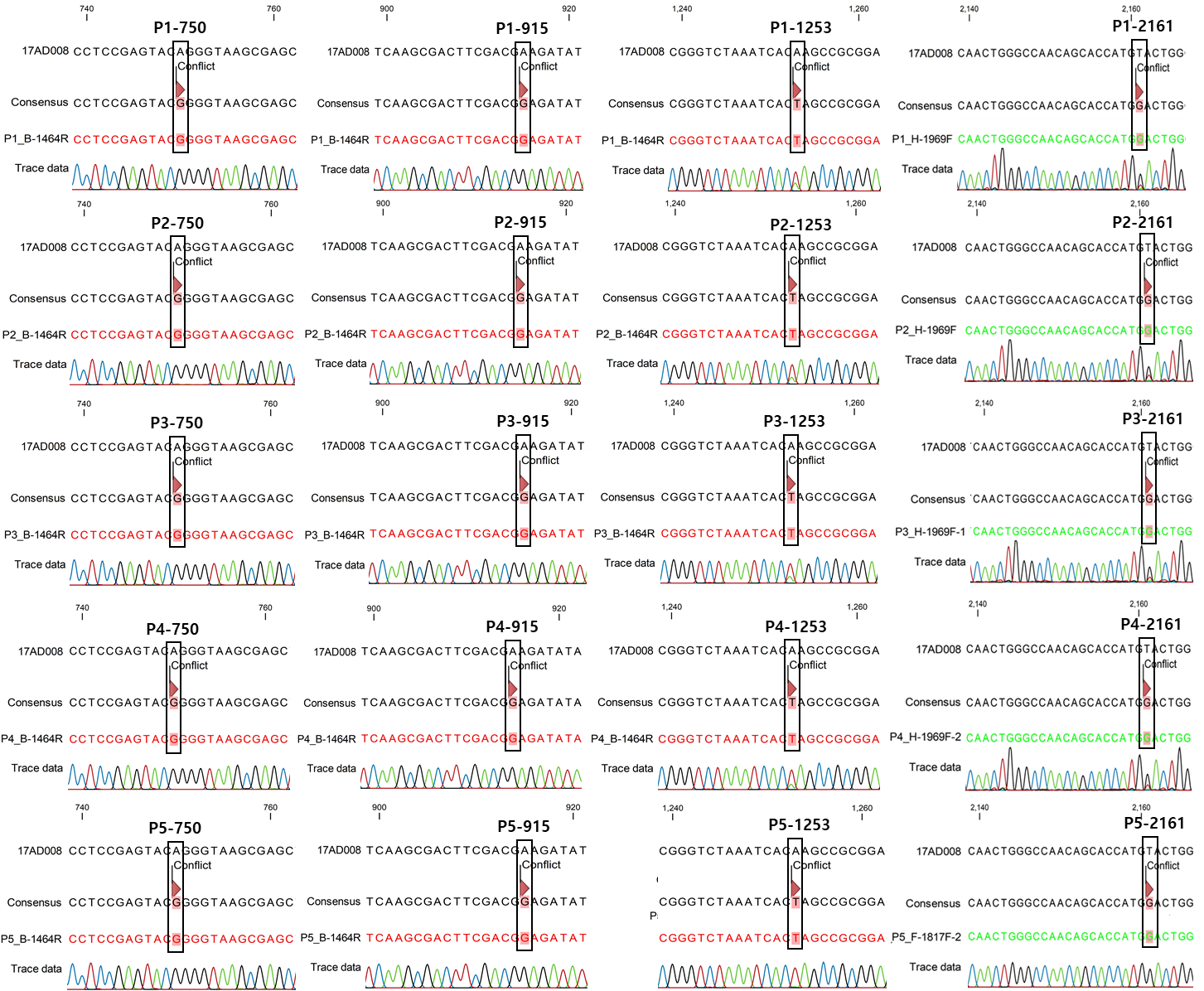


**Supplementary Fig. 4. Stability of nucleotide mutations at positions 750, 915, 1253, and 2161 in P100 after five back-passages in SPF chicks.** Comparison of nucleotide sequences of P100 before and after five consecutive back-passages in SPF chicks, confirming that the mutations at these positions remained stably fixed. P1, first back-passage; P2, second back-passage; P3, third back-passage; P4, fourth back-passage; P5, fifth back-passage.
