# Supplementary material for: A novel chicken infectious anemia virus vaccine candidate: complete attenuation, strong immunogenicity, and a built-in DIVA marker
Source: Poult Sci. 2026 May 15;105(9):107139. doi: 10.1016/j.psj.2026.107139 (PMC13226897; doi:10.1016/j.psj.2026.107139)
Supplement: Supplementary file 3 [file mmc3.docx]

Supplementary Table 2 Mean cycle threshold (Ct) values determined by qPCR, representing viral genome loads in the thymus of SPF chicks infected with P100 or WT 17AD008 CIAV strains at 3 dpi.

| Chick No. | P100 | | | WT | | | NT | | |
| --- | --- | --- | --- | --- | --- | --- | --- | --- | --- |
|  | Ct value1 | Ct value 2 | Mean of  Ct value | Ct value 1 | Ct value 2 | Mean of  Ct value | Ct value 1 | Ct value 2 | Mean of  Ct value |
| 1 | 29.68 | 30.38 | 30.03 | 29.52 | 29.21 | 29.36 | - | - | - |
| 2 | 33.00 | 33.18 | 33.09 | 31.51 | 31.72 | 31.62 | - | - | - |
| 3 | 29.26 | 29.85 | 29.55 | 30.46 | 29.89 | 30.18 | - | - | - |
| 4 | 30.27 | 30.54 | 30.41 | 31.93 | 32.79 | 32.36 | - | - | - |
| 5 | 31.91 | 31.37 | 31.64 | 28.71 | 28.76 | 28.74 | - | - | - |
